# Supplementary material for: Synthesis, Cytotoxic, and Computational Screening of Some Novel Indole–1,2,4-Triazole-Based S-Alkylated N-Aryl Acetamides
Source: Biomedicines. 2023 Nov 16;11(11):3078. doi: 10.3390/biomedicines11113078 (PMC10669176; doi:10.3390/biomedicines11113078)
Supplement: Supplementary file 1 [file biomedicines-11-03078-s001.zip › biomedicines-2686834-supplementary.pdf]

Synthesis, Cytotoxic and Computational Screening of Some Novel Indole-1,2,4-Triazole based *S*-Alkylated *N*-Aryl Acetamides

Sadaf Saeed[1], Ameer Fawad Zahoor[1,*], Azhar Rasul[2], Razia Noreen[3], Ali Irfan[1], Sajjad Ahmad[4], Shah Faisal[5], Samreen Gul Khan[1], Sami A. Al-Hussain[6], Muhammad Athar Saeed[7], Muhammed Tilahun Muhammed[8], Zeinab A. Muhammad[9], Magdi E. A. Zaki[6***]**

SUPPLEMENTARY INFORMATION

Characterization Data of Indole-1,2,4-Triazole Compounds 8a-f

2-((5-((1*H*-indol-3-yl)methyl)-4-(3,4-dichlorophenyl)-4*H*-1,2,4-triazol-3-yl)thio)-*N*-(3,4-dimethylphenyl)acetamide (8a):

Light brown powder; Yield: 69 %; m.p. 165 °C ; ^{1}H-NMR, DMSO-*d*$_6$, 400 MHz (δ/ppm): 2.16-2.18 (d, 6H, 2CH$_3$), 4.03 (s, 2H, CH$_2$), 4.13 (s, 2H, CH$_2$), 6.77-7.67 (m, 11H, aryl-H/indole-H), 10.11 (s, 1H, anilide-NH), 10.84 (s, 1H, indole-NH); ^{13}C-NMR, DMSO-*d*$_6$, 100 MHz (δ/ppm): 18.89, 19.72, 21.71, 37.48, 107.87, 111.44, 116.77, 118.22, 118.52, 120.42, 121.20, 123.67, 126.64, 127.76, 129.49, 129.70, 131.34, 131.38, 131.93, 132.79, 132.94, 136.06, 136.47, 136.55, 149.67, 155.00, 165.22. MS (ESI) m/z: 536.10 [M + 1]$^+$; Anal. Calcd. For $C_{27}H_{23}Cl_2N_5OS$: C, 60.45; H, 4.32; N, 13.05; Found; C, 60.48; H, 4.34; N, 13.03.

2-((5-((1*H*-indol-3-yl)methyl)-4-(3,4-dichlorophenyl)-4*H*-1,2,4-triazol-3-yl)thio)-*N*-(4-chlorophenyl)acetamide (8b):

Light shade rusty powder; Yield: 74 %; m.p. 130 °C. ^{1}H-NMR, DMSO-*d*$_6$, 400 MHz (δ/ppm): 4.05 (s, 2H, CH$_2$), 4.13 (s, 2H, CH$_2$), 6.76-7.67 (m, 12H, aryl-H/indole-H), 10.41 (s, 1H, anilide-NH), 10.84 (s, 1H, indole-NH); ^{13}C-NMR, DMSO-*d*$_6$, 100 MHz (δ/ppm): 21.71, 37.45, 107.86, 111.45, 118.21, 118.53, 120.74, 121.21, 123.66, 126.63, 127.19, 127.75, 128.81, 129.48, 131.34, 131.94, 132.81, 132.90, 136.06, 137.76, 149.56, 155.06, 165.77. MS (ESI) m/z: 544.03 [M + 1]$^+$; Anal. Calcd. For $C_{25}H_{18}Cl_3N_5OS$: C, 55.31; H, 3.34; N, 12.90; Found; C, 55.33; H, 3.35; N, 12.87.

2-((5-((1*H*-indol-3-yl)methyl)-4-(3,4-dichlorophenyl)-4*H*-1,2,4-triazol-3-yl)thio)-*N*-(2,4-dimethoxyphenyl)acetamide (8c):

Grey powder; Yield: 76 %; m.p. 150 °C; ^{1}H-NMR, DMSO-*d*$_6$, 400 MHz (δ/ppm): 3.67 (s, 3H, OCH$_3$), 3.76 (s, 3H, OCH$_3$), 4.08 (s, 2H, CH$_2$), 4.14 (s, 2H, CH$_2$), 6.61-7.72 (m, 11H, aryl-H/indole-H), 9.63 (s, 1H, anilide-NH), 10.83 (s, 1H, indole-NH); ^{13}C-NMR, DMSO-*d*$_6$, 100 MHz (δ/ppm): 21.72, 37.17, 55.42, 56.38, 107.46, 107.84, 108.17, 111.44, 111.98, 118.20, 118.51, 121.21, 123.66, 126.63, 127.73, 129.47, 131.34, 136.06, 143.21, 149.70, 153.03, 155.13, 166.09. MS (ESI) m/z: 568.09 [M + 1]$^+$; Anal. Calcd. For $C_{27}H_{23}Cl_2N_5O_3S$: C, 57.05; H, 4.08; N, 12.32; Found; C, 57.03; H, 4.09; N, 12.34.

2-((5-((1*H*-indol-3-yl)methyl)-4-(3,4-dichlorophenyl)-4*H*-1,2,4-triazol-3-yl)thio)-*N*-(2-methoxyphenyl)acetamide (8d):

Light yellow powder; Yield: 79 %; m.p. 169 °C; ^{1}H-NMR, $CDCl_3$, 400 MHz (δ/ppm): 3.87 (s, 3H, OCH_3), 4.04 (s, 2H, CH_2), 4.19 (s, 2H, CH_2), 6.70-7.36 (m, 12H, aryl-H/indole-H), 8.46 (s, 1H, anilide-NH), 9.55 (s, 1H, indole-NH); ^{13}C-NMR, $CDCl_3$, 100 MHz (δ/ppm): 22.02, 36.58, 56.05, 108.27, 110.37, 111.54, 118.42, 120.05, 120.26, 120.95, 122.64, 123.36, 124.42, 126.52, 127.73, 129.25, 131.54, 134.08, 136.12, 148.80, 152.23, 155.59, 165.91. MS (ESI) m/z: 538.08 $[M + 1]^+$; Anal. Calcd. For $C_{26}H_{21}Cl_2N_5O_2S$: C, 58.00; H, 3.93; N, 13.01; Found; C, 58.01; H, 3.96; N, 13.00.

2-((5-((1*H*-indol-3-yl)methyl)-4-(3,4-dichlorophenyl)-4*H*-1,2,4-triazol-3-yl)thio)-*N*-phenylacetamide (8e):

Light brown powder; Yield: 71 %; m.p. 170 °C; ^{1}H NMR, DMSO-d$_6$, 400 MHz, (δ/ppm): 4.05 (s, 2H, CH_2), 4.13 (s, 2H, CH_2), 6.77-7.67 (m, 13H, aryl-H/indole-H), 10.27 (s, 1H, anilide-NH), 10.83 (s, 1H, indole-NH); ^{13}C-NMR, DMSO-d_6, 100 MHz (δ/ppm): 21.70, 37.50, 107.85, 111.44, 118.21, 118.52, 119.20, 121.20, 123.67, 126.63, 127.63, 127.76, 128.89, 129.14, 129.49, 131.34, 131.93, 132.79, 132.92, 136.05, 138.81, 146.55, 155.02, 165.56. MS (ESI) m/z: 508.07 $[M + 1]^+$; Anal. Calcd. For $C_{25}H_{19}Cl_2N_5OS$: C, 59.06; H, 3.77; N, 13.77; Found; C, 59.08; H, 3.75; N, 13.78.

2-((5-((1*H*-indol-3-yl)methyl)-4-(3,4-dichlorophenyl)-4*H*-1,2,4-triazol-3-yl)thio)-*N*-(2-fluorophenyl)acetamide (8f)

Light yellow powder; Yield: 82 %; m.p. 161 °C; ^{1}H-NMR, $CDCl_3$, 400 MHz (δ/ppm): 4.08-4.20 (4H, $2CH_2$), 6.79-8.14 (m, 12H, aryl-H/indole-H), 8.54 (s, 1H, anilide-NH), 10.05 (s, 1H, indole-NH); ^{13}C-NMR, $CDCl_3$, 100 MHz (δ/ppm): 23.89, 38.87, 108.50, 111.48, 115.14, 115.33, 118.53, 120.11, 122.33, 122.68, 123.27, 124.47, 126.45, 126.54, 128.95, 129.24, 131.05, 131.53, 132.59, 134.09, 136.13, 146.93, 152.48, 155.71, 167.94. MS (ESI) m/z: 526.06 $[M + 1]^+$; Anal. Calcd. For $C_{25}H_{18}Cl_2FN_5OS$: C, 57.04; H, 3.45; N, 13.30; Found; C, 57.06; H, 3.43; N, 13.32.

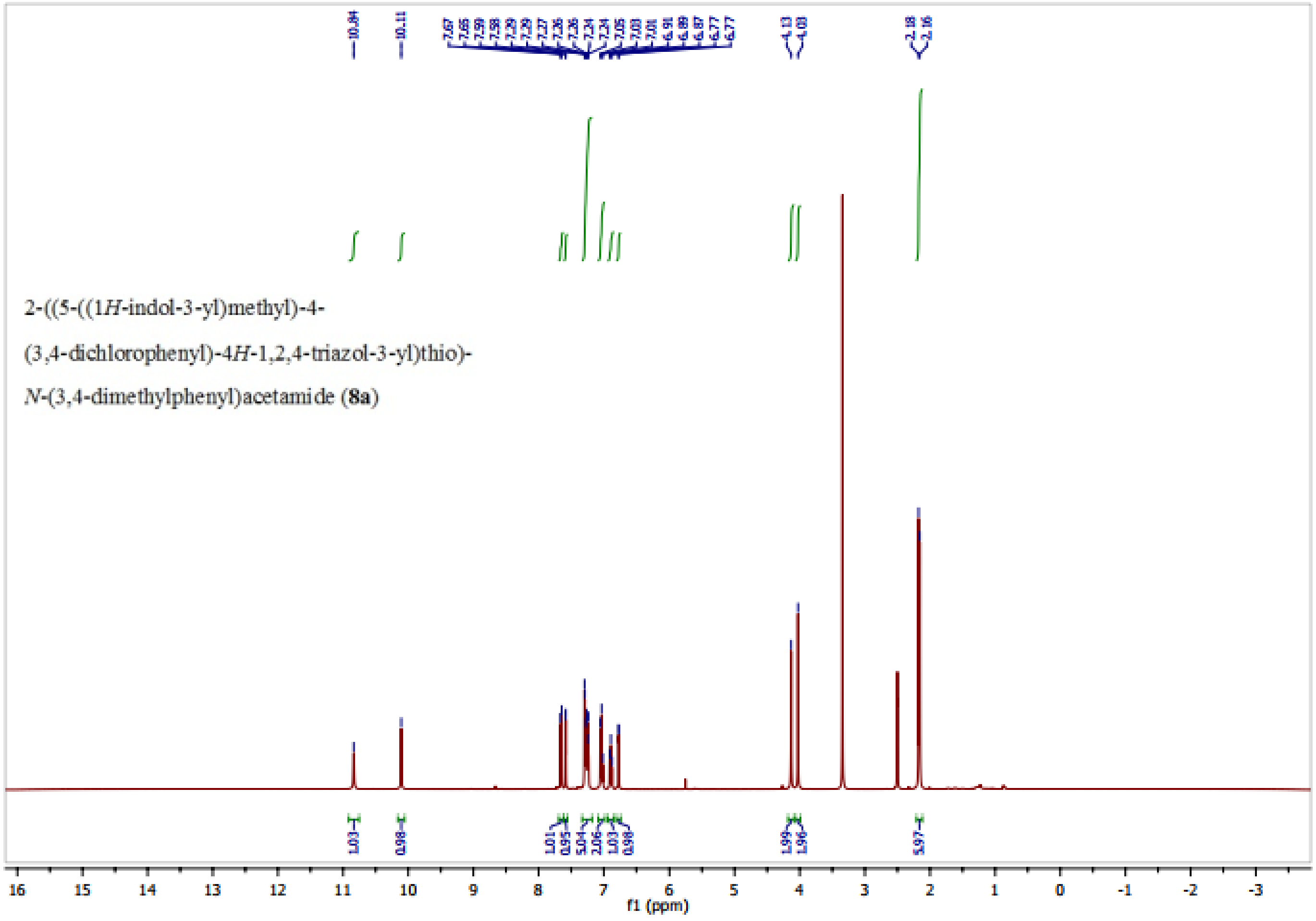

Figure S1: ^{1}H NMR Spectrum of compound **8a**.

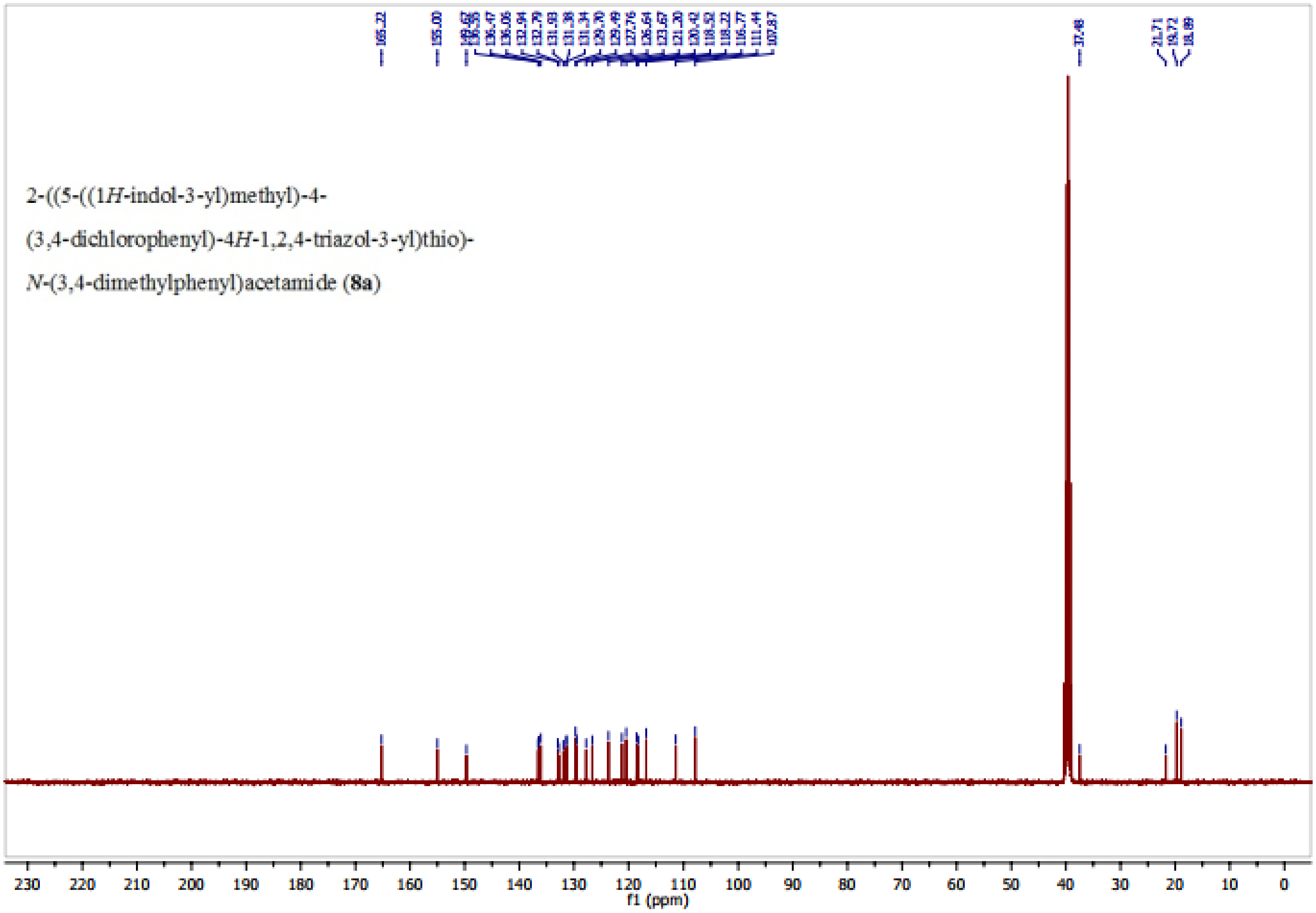

Figure S2: ^{13}C NMR Spectrum of compound **8a**.

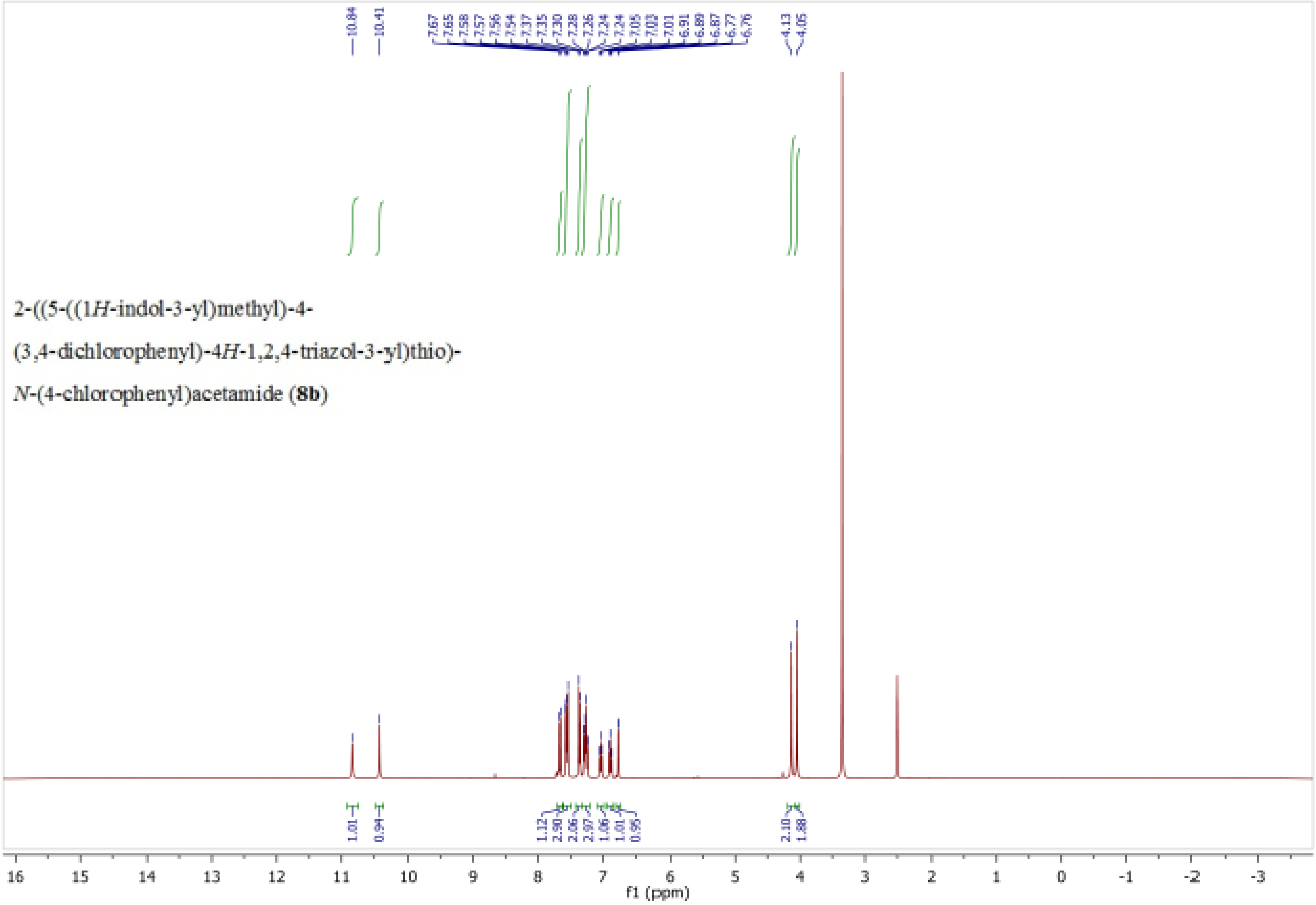

Figure S3: ¹H NMR Spectrum of compound **8b**.

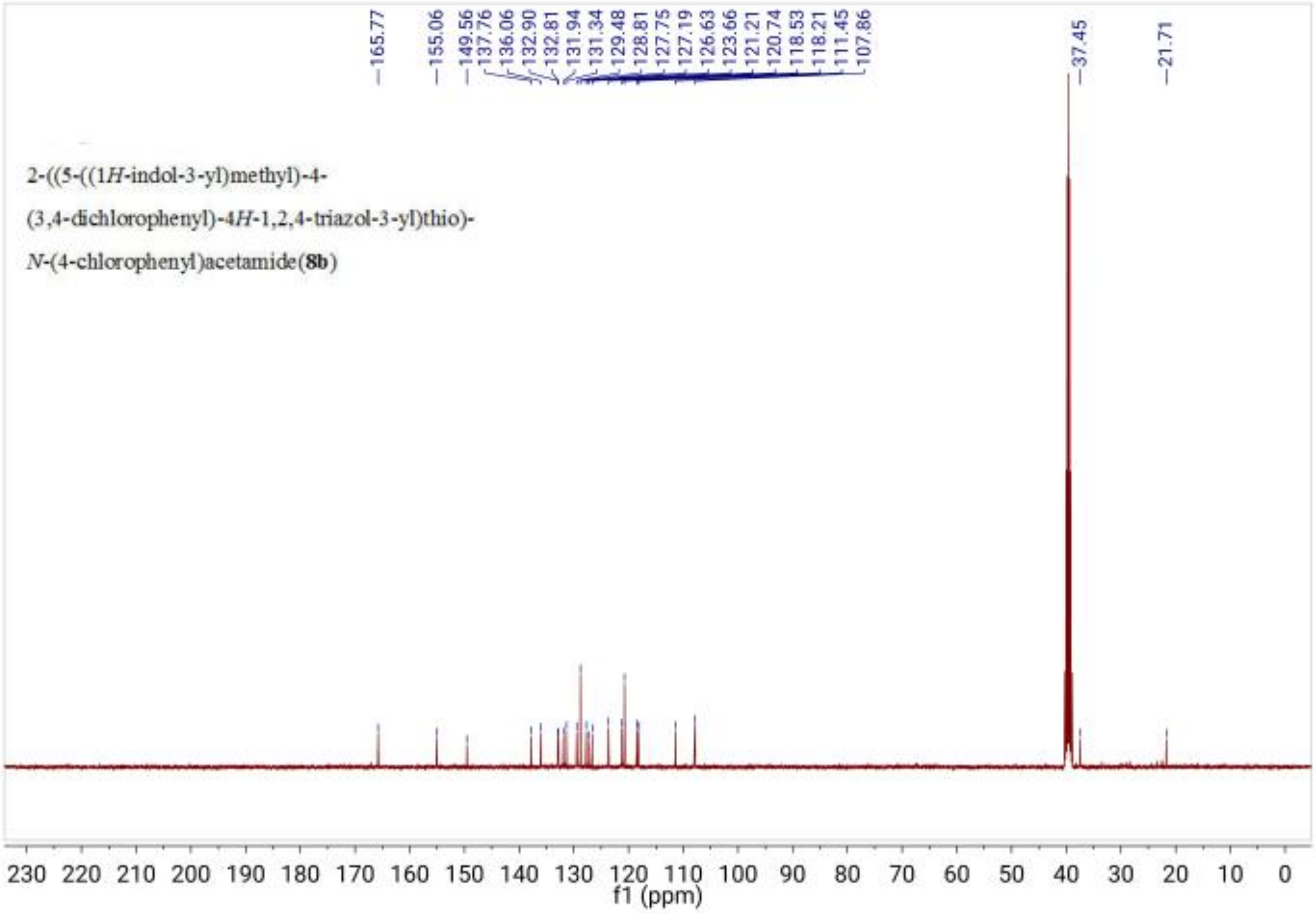

Figure S4: ^{13}C NMR Spectrum of compound **8b**.

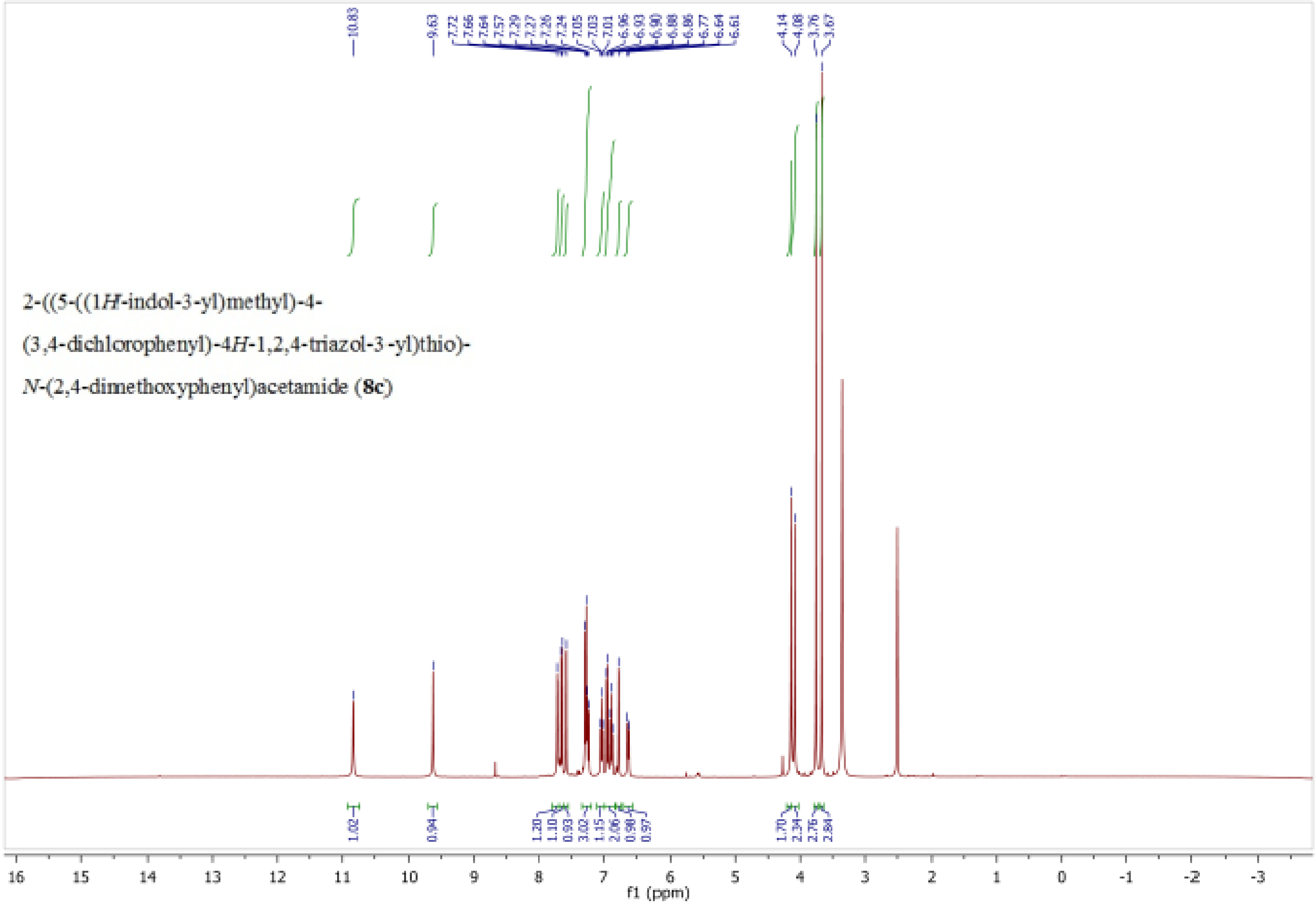

Figure S5: ^{1}H NMR Spectrum of compound **8c**.

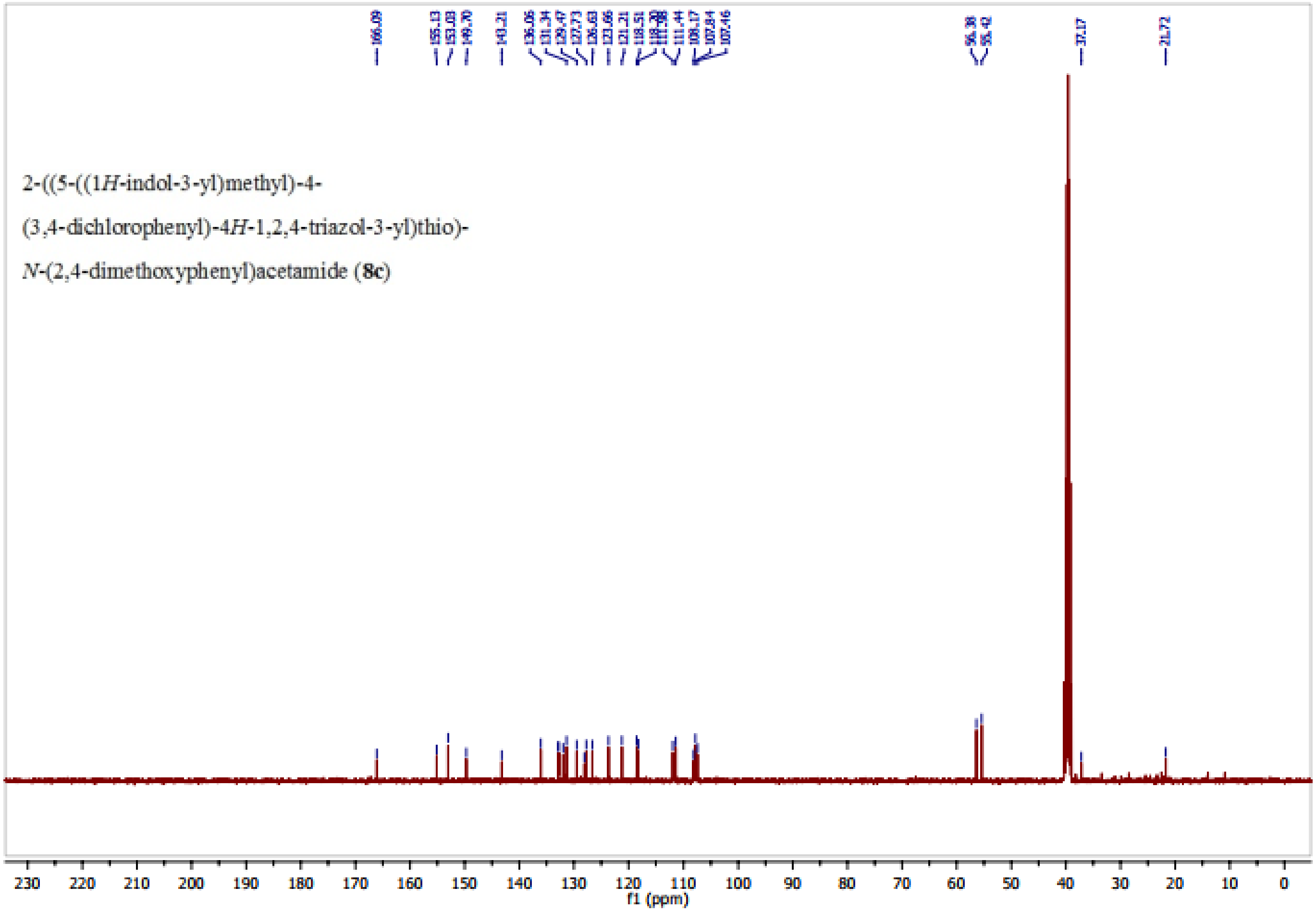

Figure S6: ^{13}C NMR Spectrum of compound **8c**.

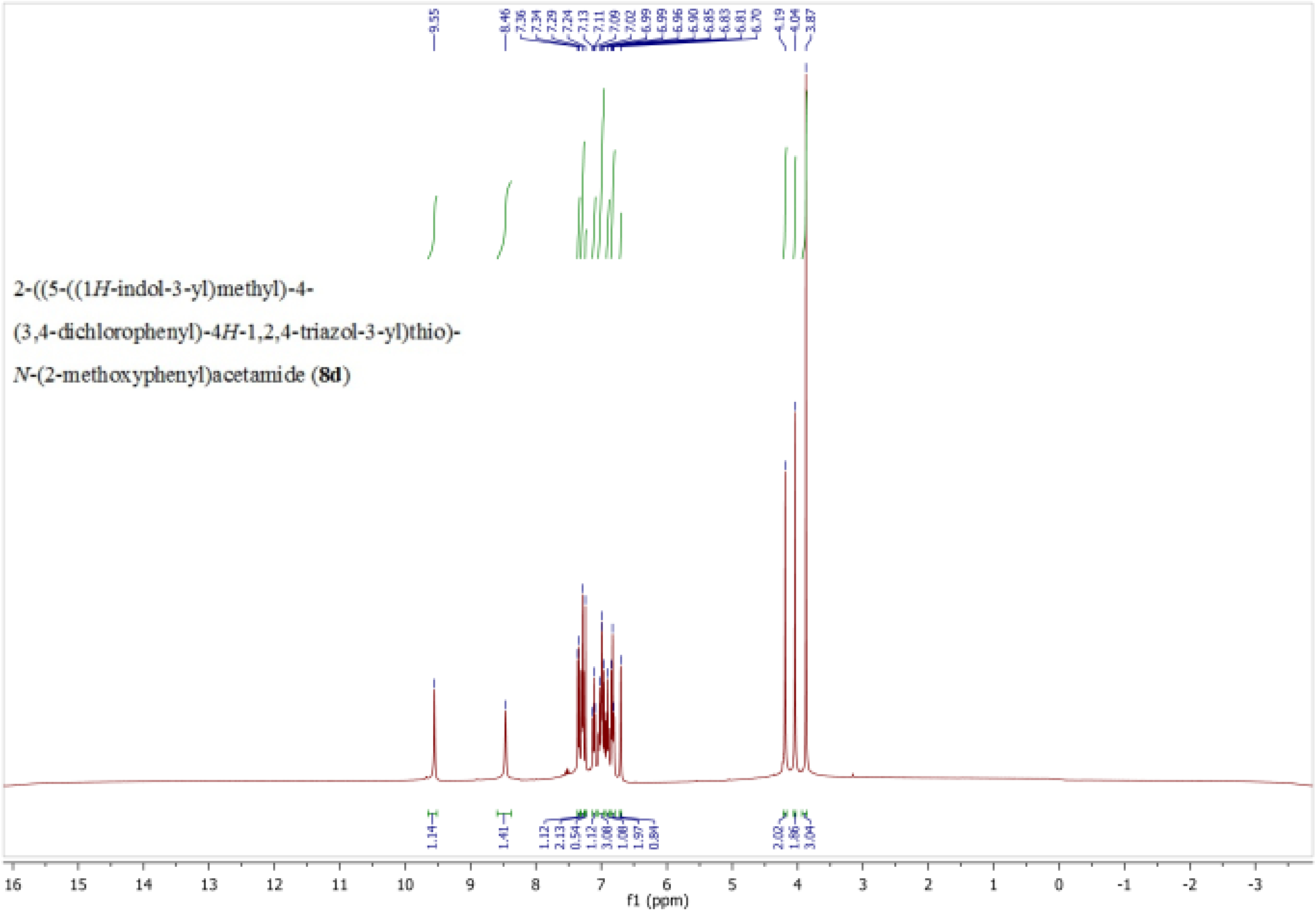

Figure S7: ^{1}H NMR Spectrum of compound **8d**.

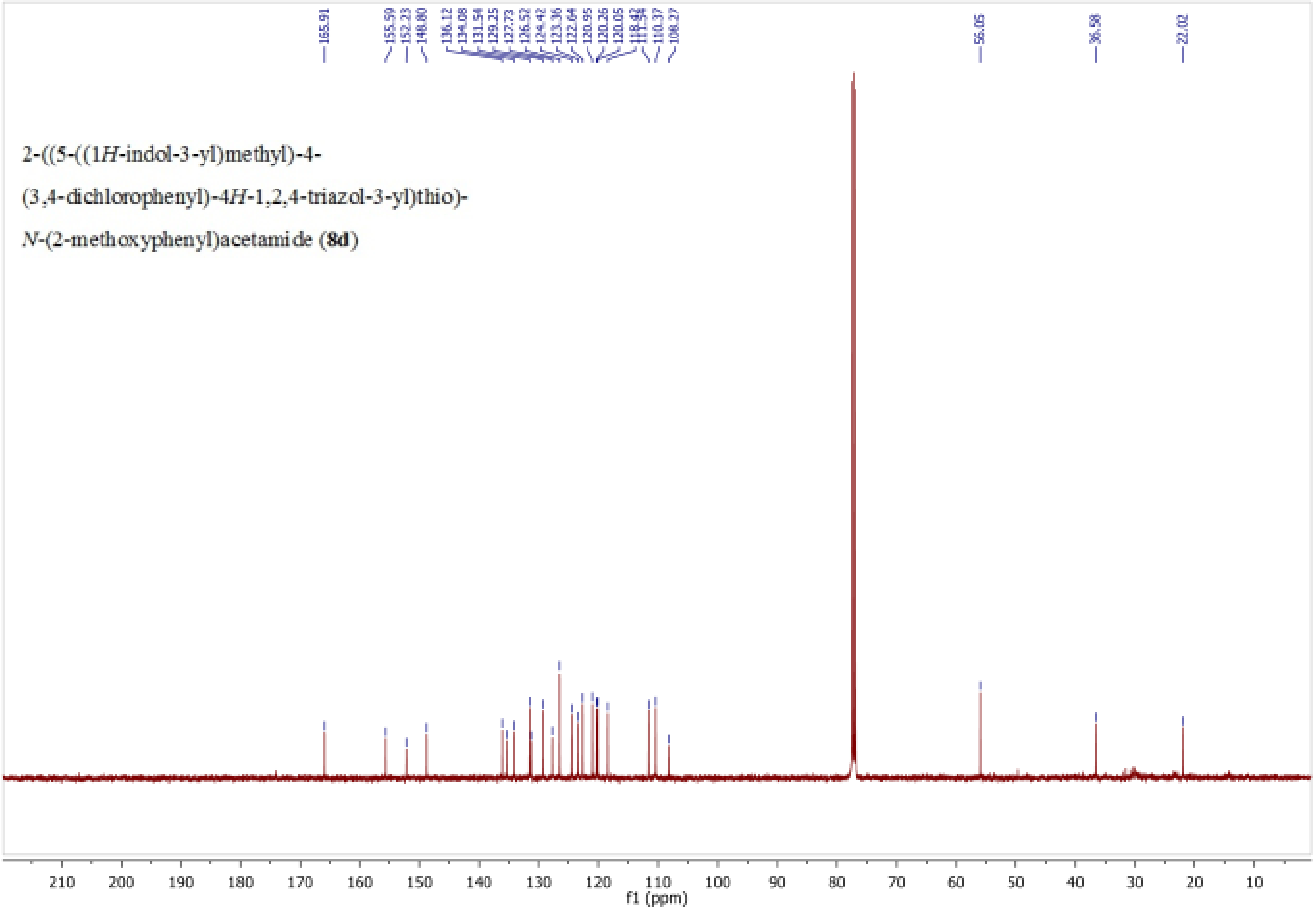

Figure S8: ^{13}C NMR Spectrum of compound **8d**.

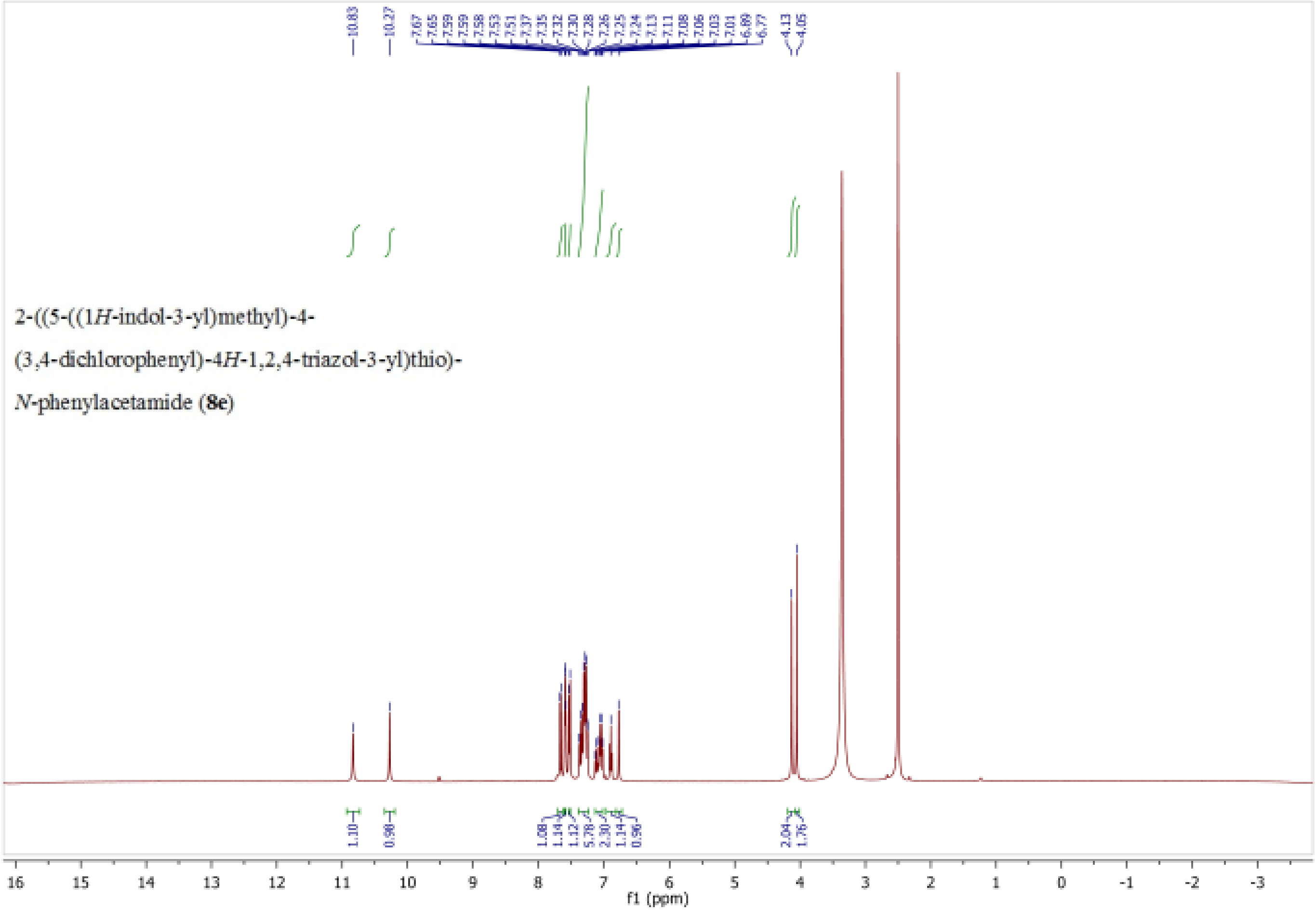

Figure S9: ^{1}H NMR Spectrum of compound **8e**.

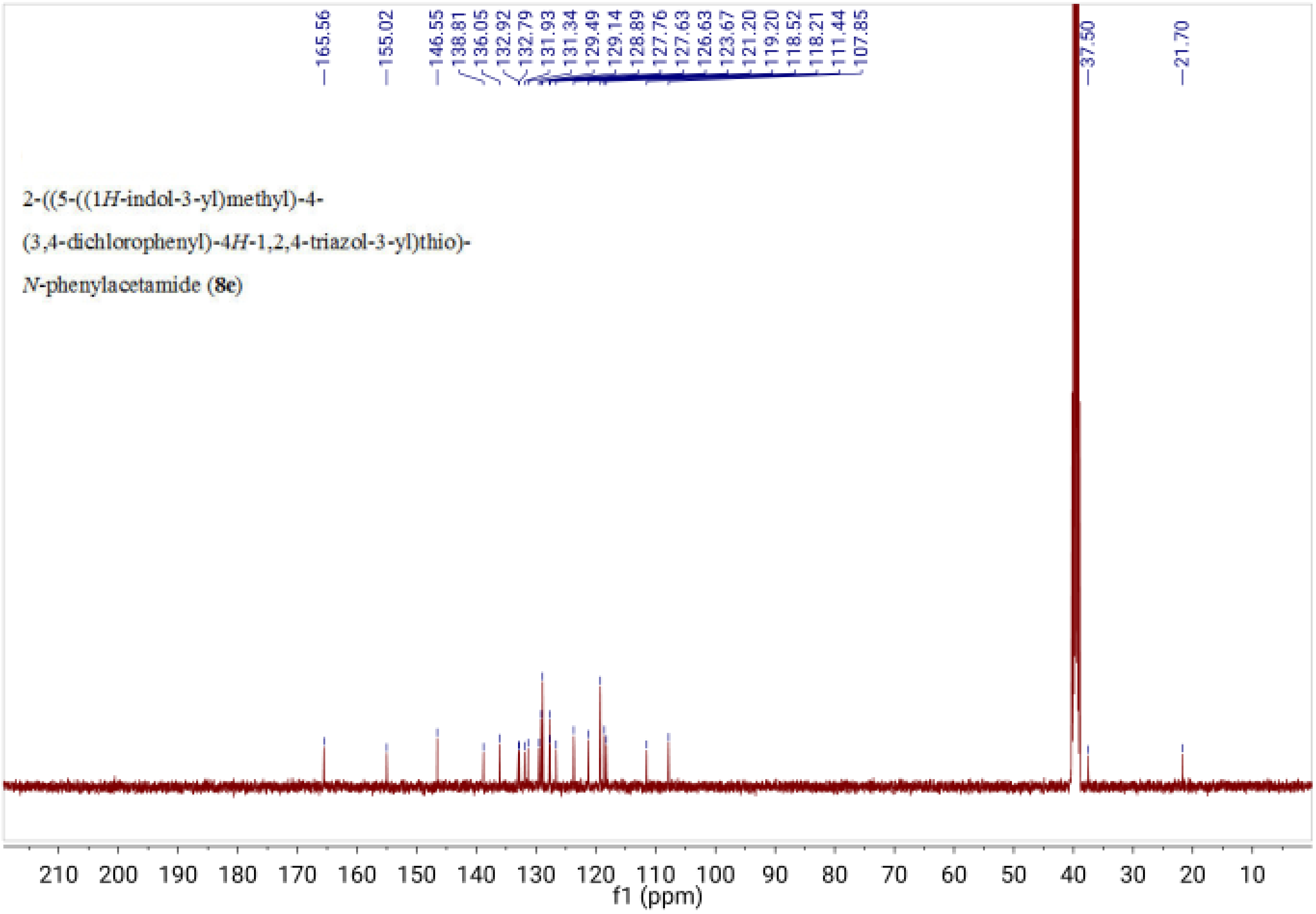

Figure S10: ^{13}C NMR Spectrum of compound **8e**.

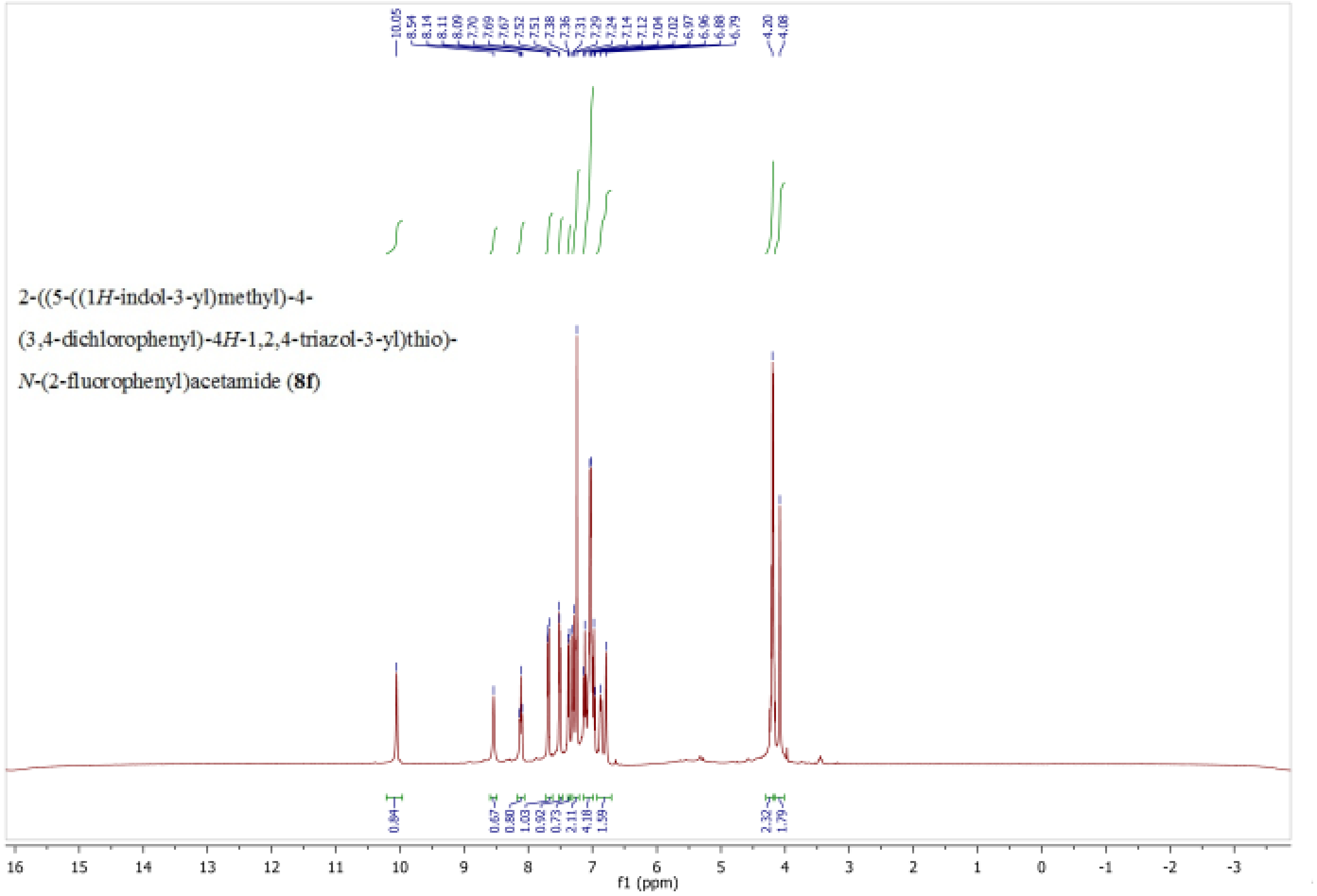

Figure S11: ^{1}H NMR Spectrum of compound **8f**.

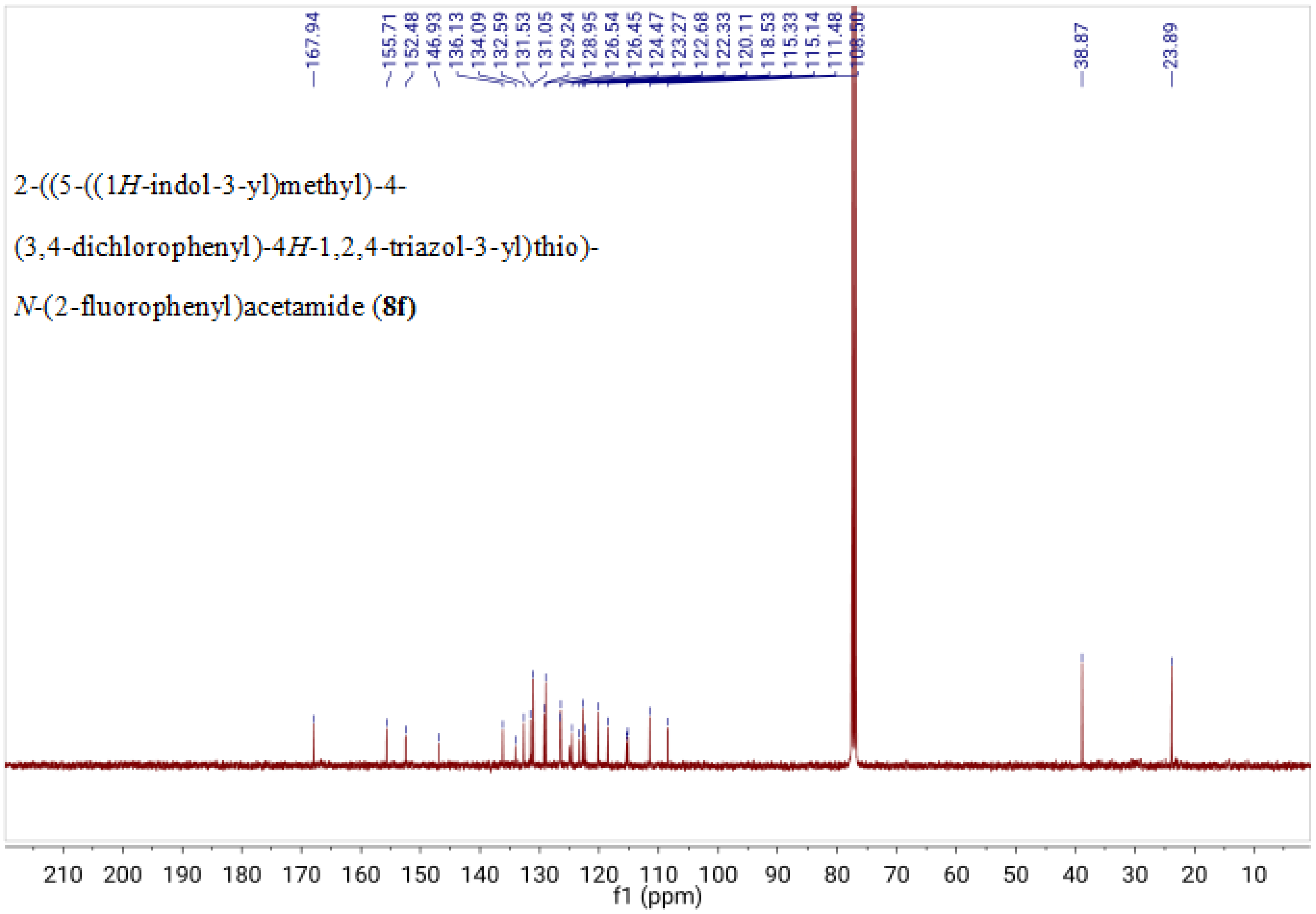

Figure S12: ^{13}C NMR Spectrum of compound **8f**.

Table S1 Binding affinities of **8a**, **8b,** and **8f** along with the control drugs of each enzyme targets

Compound's Name	PKC-θ	PI3K	VEGFR2
**8a**	–7.75 Kcal/mol	–7.50 Kcal/mol	–7.43 Kcal/mol
**8b**	–7.34 Kcal/mol	–7.59 Kcal/mol	–7.93 Kcal/mol
**8f**	–7.33 Kcal/mol	–7.33 Kcal/mol	–7.88 Kcal/mol
**Staurosporine (PKC- θ) Control**	–8.46 Kcal/mol		
**GDC-0326 (PI3K) Control**		–8.31 Kcal/mol	
**CHEMBL1254007 (VEGFR2) Control**			–8.09 Kcal/mol
